# Risk Factors Early in the 2010 Cholera Epidemic, Haiti

**DOI:** 10.3201/eid1711.110810

**Published:** 2011-11

**Authors:** Katherine A. O’Connor, Emily Cartwright, Anagha Loharikar, Janell Routh, Joanna Gaines, Marie-Délivrance Bernadette Fouché, Reginald Jean-Louis, Tracy Ayers, Dawn Johnson, Jordan W. Tappero, Thierry H. Roels, W. Roodly Archer, Georges A. Dahourou, Eric Mintz, Robert Quick, Barbara E. Mahon

**Affiliations:** Centers for Disease Control and Prevention, Atlanta, Georgia, USA (K.A. O’Connor, E. Cartwright, A. Loharikar, J. Routh, J. Gaines, T. Ayers, J.W. Tappero, T.H. Roels, W.R. Archer, E. Mintz, R. Quick, B.E. Mahon); Ministry of Public Health and Population, Port-au-Prince, Haiti (M.-D.B. Fouché); Centers for Disease Control and Prevention, Port-au-Prince (R. Jean-Louis, G.A. Dahourou); Hôpital Albert Schweitzer, Deschapelles, Haiti (D. Johnson)

**Keywords:** bacteria, enteric infections, waterborne infections, Vibrio cholerae, cholera, Haiti, diarrhea, water, sanitation, epidemic, outbreak, investigation, case control, risk factors, dispatch

## Abstract

During the early weeks of the cholera outbreak that began in Haiti in October 2010, we conducted a case–control study to identify risk factors. Drinking treated water was strongly protective against illness. Our results highlight the effectiveness of safe water in cholera control.

On October 19, 2010, the Haitian Ministry of Public Health and Population (MSPP) was notified of increased cases of acute watery diarrhea resulting in death among adults in Artibonite Department. Within 2 days, MSPP’s Laboratoire National de la Santé Publique had identified toxigenic *Vibrio cholerae* O1, serotype Ogawa, biotype El Tor in stool specimens (*1*). The first reports of illness consistent with cholera occurred on October 16, and, by November 19, cholera had reached all 10 Haitian administrative departments (*2*).

Because the first cases were in persons who worked near the Artibonite River, contaminated river water was suspected as the initial source. In a proactive effort to protect the population, MSPP rapidly implemented a cholera prevention campaign that began on October 22, 2010, to discourage the population from drinking river water, distribute water treatment products, and promote water treatment, handwashing, sanitation, and safe food preparation. To inform further prevention activities, we conducted a case–control study during the second and third weeks of the outbreak to identify risk factors for symptomatic cholera.

## The Study

This study was conducted in Artibonite Department close to where the first cases were identified. On the basis of detailed hypothesis-generating interviews with patients and known risk factors associated with cholera in other investigations in the Americas, we created a questionnaire to assess multiple exposures, including river and other water-related exposures, sanitation and hygiene practices, foods, and other factors. We enrolled and interviewed participants from October 31 through November 13, 2010, with a 4-day break during November 5–8 because of Hurricane Tomas. To rapidly generate relevant information to guide outbreak response, we set a goal of enrolling 50 case-patients and 100 controls, a sample size that, although limited, was in line with that of previous successful emergency investigations.

Eligible case-patients were persons >5 years of age who were hospitalized between October 22 and November 9 for acute watery diarrhea at the Médecins Sans Frontières cholera treatment unit in Petite Rivière, a town in a densely populated rural region near the Artibonite River. Only case-patients with the first case of acute watery diarrhea in their household since October 16 were eligible. Case-patients were interviewed about exposures during the 3 days before illness onset. Within 72 hours of the interview, we visited case-patients at home, where we observed household drinking water sources and storage containers, presence of water treatment products, access to toilet facilities, and the case-patient’s handwashing technique. Drinking water was tested for free chlorine as an objective measure of chlorine treatment. Matching by neighborhood (through a systematic door-to-door search from the case-patient’s house) and age group (5–15, 16–30, 31–45, and >46 years), we enrolled 2 controls per case-patient at the time of the visit to case-patients’ homes from households with no diarrhea since October 16. We interviewed controls about exposures during the same 3 days as the matched case-patient and made the same household observations.

The term “improved drinking water source” indicated it met the World Health Organization definition, which describes technologies that protect water from outside contamination (*3*). “Lacking safe water storage” referred to water stored in an open container or bucket without a tap. “Proper handwashing technique” was defined as observed use of soap and thorough lathering.

We performed descriptive statistical analysis and exact conditional logistic regression to compute the most likely estimate or, when small cell sizes required, the median unbiased estimate of matched odds ratios (mORs) with 95% confidence intervals (CIs). Demographic and household poverty indicators were assessed for effect modification and confounding. Matched ORs adjusting for sex and the presence of a mud floor in the household are presented in the Table. As part of the public health response to the outbreak, this investigation did not require human subjects review. Informed consent was obtained.

**Table:** Exposures of case-patients with cholera and matched controls, Artibonite Department, Haiti, October–November 2010*

Variable	No. (%) case-patients exposed, n = 49	No. (%) controls exposed, n = 98	mOR (95% CI)
Participant completed primary school†	7 (23)	18 (31)	1.0 (0.2–3.8)
Drinking water source			
Improved water source	15 (31)	23 (23)	3.5 (0.6–40.8)
Well	30 (61)	59 (60)	0.3 (0.1–2.5)
Water storage			
Lacked safe water storage	33 (79)‡	69 (74)‡	1.3 (0.5–4.0)
Bucket (unsafe storage)	31 (72)‡	67 (70)‡	1.1 (0.4–2.8)
Plastic bottle (safe storage)	7 (16)‡	19 (20)‡	0.6 (0.2–2.0)
Water treatment			
Treating drinking water before the outbreak	25 (52)‡	48 (51)‡	0.9 (0.4– 2.3)
Treating drinking water 3 d before illness onset (during outbreak)	29 (59)	82 (85)	0.2 (0.1–0.7)
Water treatment product in home	31 (69)‡	73 (75)	0.8 (0.3–2.4)
Drinking water test			
Residual chlorine presence in home drinking water >0.1 mg/L	13 (30)‡	37 (41)‡	0.4 (0.1–1.3)
Residual chlorine presence in home drinking water >0.5 mg/L	8 (16)‡	18 (18)‡	0.4 (0.1–1.8)
Contact with river water	17 (35)	26 (27)	1.1 (0.4–3.1)
Sanitation and hygiene			
Open defecation	28 (61)	40 (48)‡	2.2 (0.7–7.8)
Handwashing with soap and lather	29 (59)	20 (41)	0.6 (0.3–1.5)
Household characteristics: electricity	8 (16)	29 (30)	0.6 (0.1–2.3)
Food exposure: sugar cane juice	4 (9)‡	1 (1)‡	9.1§ (1.0–∞)

*Exposures adjusted by sex and mud floor in home. Median age of case-patients was 23 y (range 6–63 y); median age of controls was 23 y (range 5–75 y). mOR, matched odds ratio; CI, confidence interval. †Among those >15 y of age. ‡Denominators may be lower than the total number of participants because of missing data. §Median unbiased estimate.

We enrolled 49 case-patients and 98 controls; 16 (33%) case-patients and 53 (58%) controls were female. The median age was 23 years for case-patients (range 6–63 years) and controls (range 5–75 years) (Table).

Few case-patients (15/49 [31%]) or controls (23/98 [23%]) had an improved drinking water source. The most common water source was an unimproved well (30/49 [61%] of case-patients, 59/98 [60%] of controls). Similar percentages of case-patients (33/42 [79%]) and controls (69/93 [74%]) lacked safe water storage, and many case-patients (28/46 [61%]) and controls (40/84 [48%]) practiced open defecation.

Although comparable percentages of case-patients (25/48 [52%]) and controls (48/95 [51%]) reported treating their drinking water before the outbreak, case-patients were significantly less likely than controls to report treating their drinking water during the outbreak (59% vs. 85%, mOR 0.2, 95% CI 0.1–0.7). Water treatment products were found in homes of 31 (69%) of 45 case-patients and 73 (75%) of 98 controls. A lower, though not significant, percentage of case-patient households than control households (13/44 [30%] vs. 37/90 [41%]) had >0.1 mg/L of free chlorine in stored water. Among 50 foods examined, only sugar cane juice was associated with illness (9% vs.1%, mOR 9.1, CI 1.0–∞; data for other foods not shown).

## Conclusions

This study, conducted early in the cholera epidemic in Haiti in one of the first populations to be affected, demonstrated that treating drinking water was strongly protective. This finding is not unexpected, because most cholera outbreaks are spread through contaminated water, but it provides compelling specific evidence that safe drinking water is a critical need in Haiti. The disparity between the high percentage of homes with water treatment products and the lower percentage of homes with detectable chlorine in stored drinking water suggested that the communication strategy that accompanied product delivery needed modification.

The low proportions of participants with an improved water source, adequate water storage, and sanitary facilities were typical of rural Haiti (*4*). Nevertheless, the increase in reported frequency of treating drinking water during the outbreak, particularly among controls, suggested that MSPP’s cholera prevention message effectively reached at least part of the population. This campaign may have prevented the epidemic from causing even more illness and death. The association with sugar cane juice also emphasized that cholera can be transmitted by multiple routes. In the study area, sugar cane juice is typically produced by squeezing cane through a press; it is not typically made or served with water or ice, though we do not know how the juice consumed by participants was produced. After being contaminated with *V. cholerae*, however, it provides a hospitable environment for bacterial growth (*5*). These findings highlight the central importance of safe water in cholera control and the need to prevent both foodborne and waterborne transmission.

The cholera epidemic should galvanize both governmental and nongovernmental organizations to address Haitians’ need for safe water and sanitation. Experience in other cholera epidemics has shown that the benefits will likely go beyond preventing the spread of cholera; other serious public health problems, such as typhoid fever and other enteric infections, have improved substantially with effective measures to control cholera in other settings (*6*).
